# Distance Learning During the COVID-19 Lockdown and Self-Assessed Competency Development Among Radiology Residents in China: Cross-Sectional Survey

**DOI:** 10.2196/54228

**Published:** 2025-05-08

**Authors:** Peicheng Wang, Ziye Wu, Jingfeng Zhang, Yanrong He, Maoqing Jiang, Jianjun Zheng, Zhenchang Wang, Zhenghan Yang, Yanhua Chen, Jiming Zhu

**Affiliations:** 1 Vanke School of Public Health, Tsinghua University, Haidian District, Beijing, 100084, China, 86 62782199; 2 School of Medicine, Tsinghua University, Beijing, China; 3 Department of Radiology, Ningbo No. 2 Hospital, Ningbo, China; 4 Department of Radiology, Beijing Friendship Hospital, Capital Medical University, Beijing, China; 5 Department of Public Health, Policy and Systems, University of Liverpool, Liverpool, United Kingdom; 6 Institute for Healthy China, Tsinghua University, Beijing, China

**Keywords:** radiology residents, distance learning, mental health status, self-assessed competency, ACGME competencies, Accreditation Council of Graduate Medical Education

## Abstract

**Background:**

During the COVID-19 lockdown, it was difficult for residency training programs to conduct on-site, hands-on training. Distance learning, as an alternative to in-person training, could serve as a viable option during this challenging period, but few studies have assessed its role.

**Objective:**

This study aims to investigate the impact of distance learning during the lockdown on residents’ self-assessed competency development and to explore the moderating effect of poor mental health on the associations. It is hypothesized that radiology residents who were trained through distance learning during the lockdown were more likely to report higher self-assessed competency compared to those who did not receive organized, formal training.

**Methods:**

A cross-sectional survey was conducted in 2021 among all of the radiology residents in 407 radiology residency programs across 31 provinces of China. To estimate the long-term outcomes of radiology residents’ training after the initial COVID-19 outbreak, this study measured 6 core competencies developed by the US Accreditation Council for Graduate Medical Education reported by radiology residents. Multiple linear regression and moderating effect analysis were conducted to examine the associations between distance learning, mental health status, and self-assessed competencies. Mental health status moderated the association between distance learning and self-assessed competency of radiology residents.

**Results:**

A total of 2381 radiology residents (29.7% of the 8,008 nationwide) met the inclusion criteria and were included in the analysis. Among them, 71.4% (n=1699) received distance learning during the COVID-19 lockdown, and 73.2% (n=1742) reported mental health struggles ranging in severity from slight to extremely severe. Radiology residents who were trained through distance learning (β=0.35, 90% CI 0.24‐0.45) were more likely to report higher self-assessed competencies. This was particularly true for the competency of “interpersonal and communication skills” (β=0.55, 90% CI 0.39‐0.70). Whereas, the competency of “patient care and technical skills” (β=0.14, 90% CI 0.01‐0.26) benefited the least from distance learning. Poor mental health significantly moderated the relationship between distance learning and competency (β=−0.15, 90% CI −0.27 to −0.02).

**Conclusions:**

Distance learning, a means of promoting enabling environments during the COVID-19 lockdown, serves its purpose and helps generally improve residents’ self-assessed competencies, though different competency domains benefit unequally. The impact of mental health status calls for special attention so that distance learning can fulfill its potential.

## Introduction

The COVID-19 pandemic threatens the health of people globally and has brought unprecedented pressure to health systems [1,2]. The national public health system plays a vital role in fighting against pandemics by taking measurements such as surveillance and epidemiological investigations [3], case finding and management [4], and collective quarantine of close contacts [5]. However, potential challenges including insufficient alerts, low efficiency of reporting to higher authorities, and workforce shortages still exist [1]. Of note, the training of health care providers and the improvement of their professional skills have been underscored for their great significance in medical service delivery and health systems resilience [6-8].

Residency training systems serve the purpose of cultivating a qualified health workforce [9]. Standardized residency training (SRT) was initiated in 2013 in China, aiming to train doctors to meet the needs of population health [10]. With the increasing trend of competency-based medical training in global medical education, the assessment of competencies has gained ground in practice [11]. The US Accreditation Council of Graduate Medical Education (ACGME) identified 6 core competencies for physicians (ie, patient care [PC], medical knowledge [MK], system-based practice [SBP], practice-based learning and improvement [PBLI], professionalism [PROF], and interpersonal communication skill [ICS]) [11] and implemented milestones by the Next Accreditation System initiative in July 2013 [12]. Residency education and competency-based practice assessed by the milestones are common requirements of ACGME and have been used in residency training in China [9,13].

COVID-19 has changed medical education dramatically, especially during the lockdown period. The impacts of COVID-19 on medical education in radiology, surgery, and emergency medicine have gained attention [14-16]. Radiology is related to other medical specialties and all levels of health care delivery [17]. Radiology residents were typically required to rotate between different specialties to obtain knowledge and clinical skills [18]. The mandatory social distancing challenged the traditional training on radiology trainee approaches such as teaching at workstations [19]. In China, SRT in radiology spans 3 years and involves workstation-based training throughout rotations in various specialties and departments [9]. In the first year, residents undergo rotations in the departments of radiology, ultrasound medicine, nuclear medicine, pathology, and relevant clinical departments. In the second and third years, they receive advanced rotational training within radiology subspecialties such as computed tomography, magnetic resonance imaging, x-ray, and interventional radiology [9]. Due to COVID-19, the mode of residency training has been switched from traditional in-person classes to distance learning [20], posing challenges to the effectiveness of rotational training and the developing competency of residents.

Numerous benefits have been found for distance learning. For instance, residents can schedule more flexibly and access the courses more easily [21]. They can learn at their own pace with the help of recorded lectures and communicate with professionals and peers on the web at their own convenience [22]. The positive acceptance and a higher level of satisfaction with distance learning have been reported by residents in Canada and the United States [21,23]. However, the practice of digital readout in distance learning is similar to the experience of in-person reading, in addition to the difficulties of gauging body language during practical operations or in the use of medical instruments [24], which may lead to unsatisfactory outcomes in radiology education. Meanwhile, the COVID-19 pandemic has impacted the mental health status of health care professionals dramatically [25]. Students who experienced distance learning during the pandemic had been found to have psychological distress [26,27]. According to the Job Demands-Resources model, mental health is a personal resource that helps residents to deal with job challenges by moderating their performance [28-31]. Accordingly, it is of great importance to take care of the mental health of residents who have experienced distance learning [32]. To date, during the COVID-19 lockdown, when workstation-based training was difficult to deliver, the role of distance learning remains unclear. It is also uncertain whether psychological status affects the effectiveness of distance learning.

In sum, the COVID-19 lockdown had brought substantial challenges to radiology training programs, which had to transition from face-to-face instruction to remote learning. In the meantime, mental distress caused by factors such as social distancing may make residency training rather difficult. Given that distance learning remains a primary alternative when traditional teaching is not feasible (such as during pandemic outbreaks and lockdowns), yet few studies have explored its effects, we aimed to investigate the impact of distance learning on the development of self-assessed competencies as well as the moderating effect of mental health status. We hypothesize that radiology residents who received distance learning during the COVID-19 lockdown were more likely to report higher self-assessed competencies compared to those who did not receive organized, formal training during the same period (ie, nondistance learners) and that this association was moderated by poor mental health. To test this, we used a nationwide survey dataset of radiology residents in China, which collected information on distance learning and mental health status during the lockdown (January-May 2020), and self-assessed competencies 6 months later. Previous studies have shown a strong positive correlation between the assessments by Clinical Competency Committees and residents’ self-evaluations using the milestones. This suggests that residents are generally able to accurately assess their own competencies, which in turn supports the validity of using milestone assessments as an effective measure of self-assessed competency in this study [33].

## Methods

### Ethical Considerations

The study was conducted according to the guidelines of the Declaration of Helsinki, and approved by the institution review board of Tsinghua University, China (20210140). Informed consent was obtained before the research started, and data were deidentified to ensure participant privacy. Participation in this survey was voluntary, participants did not receive any incentives to take part in the study.

### Study Setting and Population

A nationwide retrospective cross-sectional survey was conducted on the web by the Chinese Association of Radiologists (CAR) during December 1, 2020, to April 30, 2021, targeting all the radiology residents in 407 radiology residency programs across 31 provinces of China.

To complete the distribution of the questionnaire and to ensure the participation of the radiology residents, we contacted the directors of the targeted hospitals’ radiology departments by email or telephone to inform them of the purpose and details of the survey initiated by the CAR. The directors were then instructed to share the link of the questionnaire posted on the popular web-based survey platform “Wenjuanxing” with radiology residents. Anonymous responses to the questionnaire were submitted. All participants were informed that the questionnaire could only be filled out once, that participation was completely voluntary, and that they could withdraw at any time without penalty. During the 5-month survey period, the research team cooperated well with the CAR for the monitoring of the participation. If there was a low rate of submission, the CAR would require the hospital to improve the quality and quantity of their survey response. This proactive approach could help increase the number of responses and the representativeness of the population. Residents who did not undergo residency training during the COVID-19 epidemic between January and May 2020 would be excluded.

### Measures and Outcomes

#### Distance Learning

Distance learning was asked “Was your training institution changed the teaching arrangements to the form of distance learning to reduce the negative impact during the initial COVID-19 outbreak?” The response options were either “yes or no.” In contrast, nondistance learning participants (ie, nondistance learners) were those who did not receive organized, formal training during the same period. They primarily stayed at home on leave, reported daily health status, and engaged in delayed teaching plans or self-directed learning.

#### Mental Health Status

Mental health status was measured by the question “Did you suffer psychological distress during the initial COVID-19 outbreak? (from January to May 2020 in China).” A 5-point Likert scale was used to measure the degree of mental health struggles: 1=no impact, 2=mild impact, 3=moderate impact, 4=severe impact, and 5=extremely severe impact. Radiology residents can choose any rating between 1 and 5 (single choice). The variable is correlated with long-term mental health (depression and burnout) measured by the Depression and Anxiety Stress Scale—Depression and Maslach Burnout Inventory scales (Multimedia Appendix 1). The Cronbach α reliability coefficient for depression and burnout was 0.930 and 0.957, respectively.

#### Self-Assessed Competency

Milestone-based assessment of competencies for residence is one of the common requirements of the ACGME [11]. Self-assessment plays a key role in this process by fostering reflection on professional actions, identifying learning needs, and enabling residents to develop and refine personalized improvement plans [34]. Moreover, residents’ self-assessments showed a strong alignment with the Clinical Competency Committee evaluations across postgraduate year levels [33,35,36]. To estimate the long-term outcomes of radiology residents’ training after the initial COVID-19 outbreak, our study measured 6 core competencies developed by the ACGME that were assessed by radiology residents themselves. As is suggested by the experts, we selected 9 subcompetencies from diagnostic radiology milestones to represent the 6 ACGME core competencies: 2 PC subcompetencies, 2 MK subcompetencies, 2 PBLI subcompetencies, 1 SBP subcompetency, 1 PROF subcompetency, and 1 ICS subcompetency.

**Figure 1.**
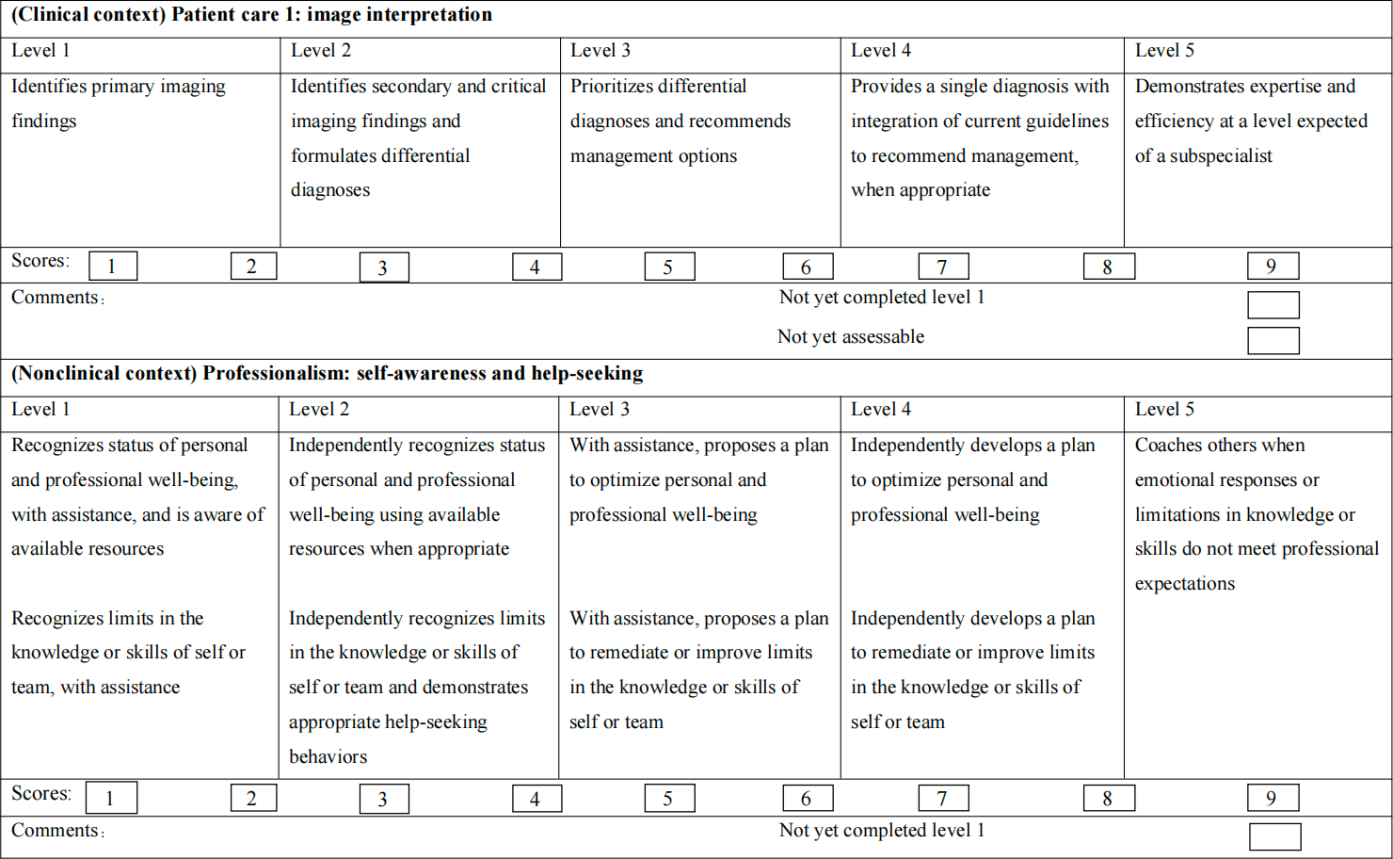
Milestone sets for patient care 1 (image interpretation) and professionalism (self-awareness and help-seeking).

A dedicated section of the questionnaire is designed to assess 9 subcompetencies with 9 single-choice questions. Radiologists are able to select a score ranging from 0 to 9 for each competency. Examples of milestone sets for each subcompetency are shown in Figure 1. The primary outcome was self-evaluation milestone (SEM) scores (range 0‐9 scores) for 9 subcompetencies and the average SEM scores.

#### Sociodemographic Characteristics

The sociodemographic information included age (≤27 or >27 years), sex (male or female), educational level (bachelor’s degree or master’s degree or above), training year (the second year or the third year), training sites level (grade-a tertiary general hospital, grade-a tertiary specialized hospital, grade-b tertiary general hospital, or others), undergraduate major (clinical medicine, medical imaging, or others), working hours per week (≤40, 40‐48, or >48), annual after-tax income in 2020 (analyzed as continuous variable), and types of residents (professional master or nonprofessional master).

### Statistical Analysis

The SEM scores of radiology residents for 9 subcompetencies were reported by means and SDs). The differences in SEM scores of residents between distance learning and nondistance learning were compared using independent samples 2-tailed *t* test. To explore the association between distance learning and competencies, multiple linear regression (MLR) models were constructed. The dependent variables were SEM scores of 9 subcompetencies, and the key explanatory variable was distance learning. The moderating effect of mental health on distance learning was explored by the MLR model. The significance of the moderating effect was tested by simple slope analysis. All models were controlled for participants’ characteristics. A variance inflation factor was used to detect the multicollinearity of independent variables for all models (variance inflation factor scores <3). A *P* value of <.05 was considered statistically significant for 2-tailed tests (*t* test or chi-square test). A conservative level of *P* value of <.10 was used to assess potential moderators in the regression, which was reported by a coefficient (β) and 90% CIs [37,38]. All statistical analyses were performed by STATA (version 17.0; StataCorp LLC).

## Results

### Participants’ Characteristics

**Figure 2.**
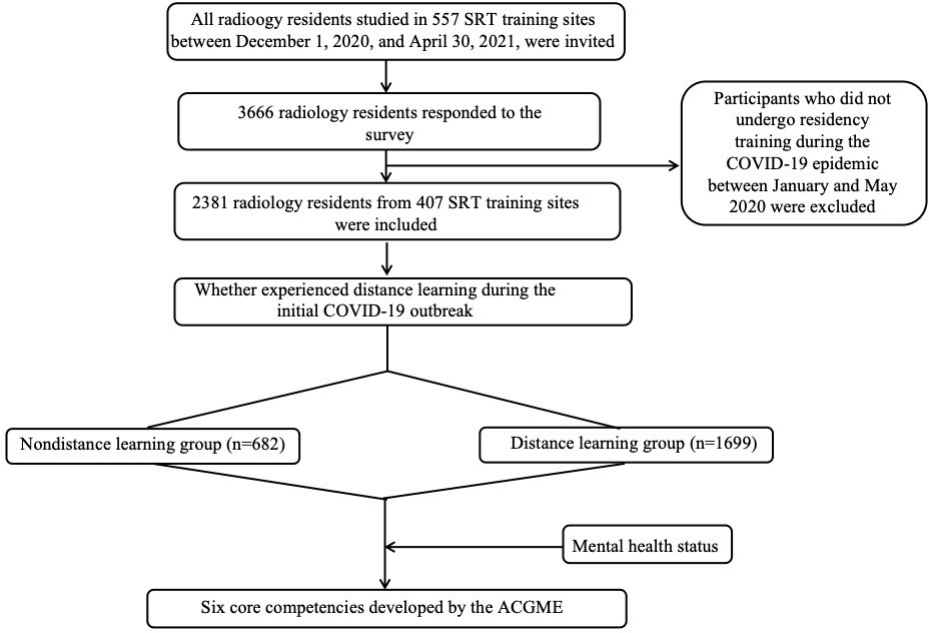
Flowchart of the study design. ACGME: Accreditation Council for Graduate Medical Education; SRT: standardized residency training.

Of the 8008 targeted radiology residents, 2381 (overall effective response rate: 29.7%) participated in this survey (Figure 2). As is shown in Table 1, the mean age of the participants was 27.8 (SD 2.4) years. In total, 58.5% (n=1392) of them were female, and 50.5% (n=1202) were in the third-year training. The majority of the participants received training in a grade-a tertiary hospital (n=2310, 97%), had a bachelor’s degree (n=2187, 91.9%), and their undergraduate major was medical imaging (n=2016, 84.7%). The median annual after-tax income was 40,000 RMB (IQR 10,000‐60,000; a currency exchange rate of 1 RMB=US $0.145 is applicable), with 25.8% (n=614) of them earning more than 60,000 RMB (about US $ 8698.9). The average working hours per week was 44.3 (SD 12.5) hours, and 23.1% (n=551) of the participants worked more than 48 hours per week. During the initial COVID-19 outbreak from January 2020 to May 2020, 71.4% (n=1699) of the radiology residents participated in distance learning, and 73.2% (n=1742) of them reported slight or severe mental health struggles. In total, 35.8% (n=853) of the participants contributed to the prevention and control of COVID-19.

**Table 1.** Characteristics of participants in China.

Variables	Total (N=2381)	Distance learning		*P* value
		Yes (n=1699)	No (n=682)	
Region, n (%)				.36
East	945 (36.7)	663 (70.2)	282 (29.9)	
Central	496 (20.8)	358 (72.2)	138 (27.8)	
West	788 (33.1)	561 (71.2)	227 (28.8)	
Northeast	152 (6.4)	117 (77)	35 (23)	
Age (years), mean (SD)	27.8 (2.4)	27.8 (2.4)	27.7 (2.4)	.28
≤27, n (%)	1293 (54.3)	915 (70.8)	378 (29.2)	.49
>27, n (%)	1088 (45.7)	784 (72.1)	304 (27.9)	.49
Sex, n (%)				.02
Male	989 (41.5)	680 (68.8)	309 (31.2)	
Female	1392 (58.5)	1019 (73.2)	373 (26.8)	
SRT^a^ training years, n (%)				.63
Second year	1179 (49.5)	836 (70.9)	343 (29.1)	
Third year	1202 (50.5)	863 (71.8)	339 (28.2)	
SRT sites level, n (%)				.34
Grade-A tertiary general hospital	2310 (97)	1649 (71.4)	661 (28.6)	
Grade-A tertiary specialized hospital	51 (2.1)	39 (76.5)	12 (23.5)	
Grade-B tertiary general hospital	15 (0.6)	8 (53.3)	7 (46.7)	
Others	5 (0.2)	3 (60)	2 (40)	
Education level, n (%)				.55
Bachelor’s degree	2187 (91.9)	1557 (71.2)	630 (28.8)	
Master’s or doctoral degree	194 (8.2)	142 (73.2)	52 (26.8)	
Undergraduate major, n (%)				.12
Clinical medicine	346 (14.5)	238 (68.8)	108 (31.2)	
Medical imaging	2016 (84.7)	1444 (71.6)	572 (28.4)	
Others	19 (0.8)	17 (89.5)	2 (10.5)	
Type of residents, n (%)				.007
Professional master	774 (32.5)	580 (74.9)	194 (25.1)	
Nonprofessional master	1607 (67.5)	1119 (69.6)	488 (30.4)	
Annual after-tax income (RMB^b^), median (IQR)	43,800(10,000-67,000)	42,700(9600-60,000)	46,600(10,000-70,000)	.02
≤10,000, n (%)	691 (29)	513 (74.2)	178 (25.8)	.12
10,001‐40,000, n (%)	565 (23.7)	400 (70.8)	165 (29.2)	.12
40,001‐60,000, n (%)	511 (21.5)	367 (71.8)	144 (28.2)	.12
>60,000, n (%)	614 (25.8)	419 (68.2)	195 (31.8)	.12
Working hours per week (hours), mean (SD)	44.3 (12.5)	43.9 (11.8)	45.3 (14.0)	.01
≤40, n (%)	1311 (55.1)	948 (72.3)	363 (27.7)	.43
41‐48, n (%)	519 (21.8)	369 (71.1)	150 (28.9)	.43
>48, n (%)	551 (23.1)	382 (69.3)	169 (30.7)	.43
Mental health impact during the initial COVID-19 outbreak, n (%)				<.001
No impact	639 (26.8)	469 (73.4)	170 (26.6)	
Mild impact	1249 (52.5)	903 (72.3)	346 (27.7)	
Moderate impact	397 (16.7)	276 (69.5)	121 (30.5)	
Severe impact	79 (3.3)	45 (57)	34 (43)	
Extremely severe impact	17 (0.7)	6 (35.3)	11 (64.7)	
COVID-19–related work participation, n (%)				.90
Yes	853 (35.8)	610 (71.5)	243 (28.5)	
No	1528 (64.2)	1089 (71.3)	439 (28.7)	

a SRT: standardized residency training.

b A currency exchange rate of 1 RMB=US $0.145 is applicable.

### SEM Scores of Radiology Residents Between Distance Learning and Nondistance Learning

The mean score of competencies and a comparison of subcompetencies scores were presented in Table 2. The overall average score of radiology residents’ competency was 3.37 (SD 1.47). The average score of participants who received distance learning was 3.46 (SD 1.49), higher than those who did not (mean 3.13, SD 1.39; *P*<.001). Residents who received distance learning outperformed in all subcompetencies than those without distance learning during the initial COVID-19 outbreak (PC-1: *P*=.007; MK-1: *P*=.004; and others: *P<*.001), except for PC-2 (*P*=.09; Table 2). Due to the low response rate, Mann-Whitney tests were performed. The results were similar (Multimedia Appendix 2). For radiology residents who had not participated in COVID-19–related activities (1528/2381; Multimedia Appendix 3), the differences between residents’ competencies or subcompetencies showed the same trends, except for the PC-1 (P=.10).

**Table 2.** Self-evaluation milestone scores for radiology residents between distance learning and nondistance learning.

Diagnostic radiology subcompetencies	Total (N=2381), mean (SD)	Distance learning		*P* value
		Yes (n=1699), mean (SD)	No (n=682), mean (SD)	
PC^a^				
PC-1: image interpretation	3.90 (1.69)	3.96 (1.68)	3.75 (1.72)	.007
PC-2: competence in procedures	2.25 (1.77)	2.28 (1.81)	2.16 (1.65)	.09
MK^b^				
MK-1: diagnostic knowledge	3.75 (1.75)	3.82 (1.76)	3.59 (1.73)	.004
MK-2: imaging technology and image acquisition	3.53 (1.90)	3.62 (1.92)	3.29 (1.81)	<.001
SBP^c^				
SBP-1: system navigation for patient-centered care	2.86 (1.88)	2.96 (1.90)	2.61 (1.80)	<.001
SBP-2: contrast agent safety	3.57 (1.95)	3.71 (1.98)	3.21 (1.80)	<.001
PBLI^d^				
PBLI: evidence-based and informed practice	3.25 (1.84)	3.34 (1.86)	3.02 (1.78)	<.001
PROF^e^				
PROF: self-awareness and help-seeking	3.49 (1.90)	3.61 (1.92)	3.20 (1.83)	<.001
ICS^f^				
ICS: patient- and family-centered communication	3.72 (2.10)	3.87 (2.11)	3.33 (2.03)	<.001
Average (all subcompetencies)	3.37 (1.47)	3.46 (1.49)	3.13 (1.39)	<.001

a PC: patient care.

b MK: medical knowledge.

c SBP: system-based practice.

d PBLI: practice-based learning and improvement.

e PROF: professionalism.

f ICS: interpersonal communication skill.

### Association Between Distance Learning and Competencies Among Radiology Residents

**Figure 3.**
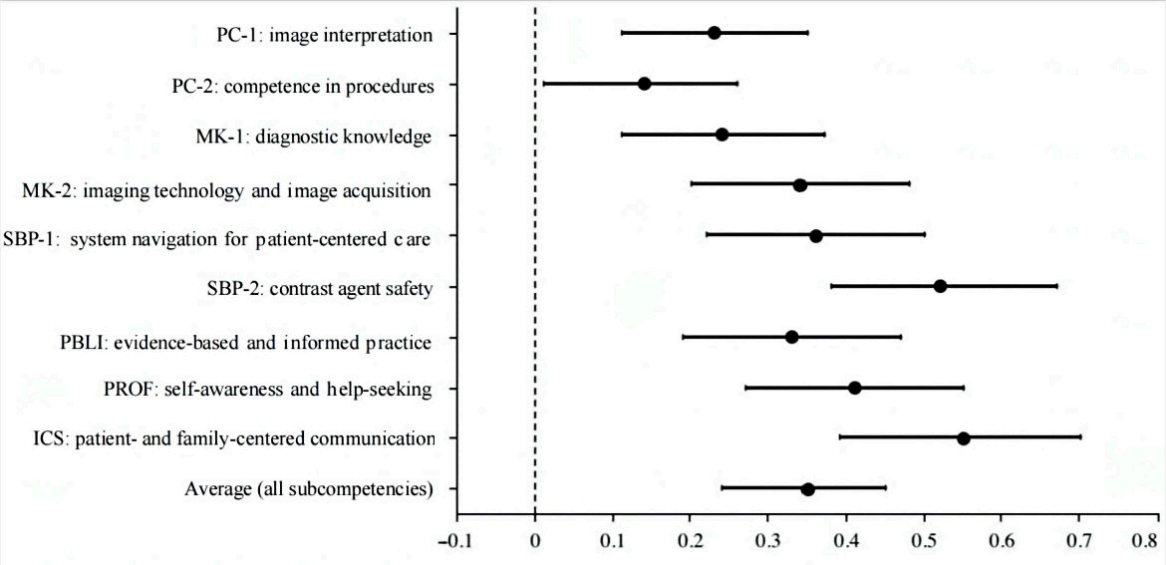
Associations between distance learning and competencies among residents. ICS: interpersonal communication skill; MK: medical knowledge; PBLI: practice-based learning and improvement; PC: patient care; PROF: professionalism; SBP: system-based practice.

As is shown in Figure 3 (see also Multimedia Appendix 4), MLR analyses showed that radiology residents who were trained through distance learning were more likely to report high competencies (β=0.35, 90% CI 0.24‐0.45) after adjusted by participants’ characteristics, including age, sex, educational level, training years, working hours per week, annual after-tax income in 2020, and types of residents. This was particularly evident in the competencies of “interpersonal and communication skills” (β=0.55, 90% CI 0.39‐0.70) and “contrast agent safety” (β=0.52, 90% CI 0.38‐0.67). Whereas, the competency of “patient care and technical skills” benefited the least from distance learning (β=0.14, 90% CI 0.01‐0.26). The effect of each explanatory variable on the overall average SEM score is shown in Multimedia Appendix 5. Factors associated with higher competencies included older age (>27 years: β=0.12, 90% CI 0.01‐0.22), being male (β=0.28, 90% CI 0.18‐0.37), and having a longer SRT training year (third year: β=0.51, 90% CI 0.41‐0.61).

### The Moderating Effect of Mental Health Status Between Distance Learning and Competencies

**Figure 4.**
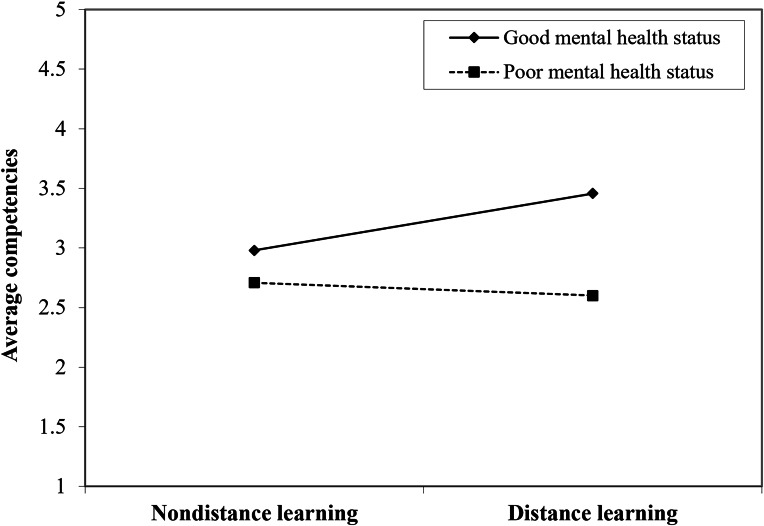
The moderating effect of mental health on the relationship between distance learning and average competencies. Two lines are the visual representation of different moderation effects of distance learning on competencies when the mental health status is at its +1SD and –1SD.

The association between mental health status and competencies is shown in Multimedia Appendix 6. We controlled the covariates mentioned earlier to investigate the moderating effect of mental health on the association between distance learning and competencies. As is shown in Table 3, there was a significant association between distance learning and radiology residents’ competencies (β=0.63, 90% CI 0.34‐0.91; *P*<.001) and was moderated by mental health status (β=–0.15, 90% CI −0.27 to −0.02; *P*=.06). The relationship between distance learning and competencies at low and high (mean−SD and mean + SD, respectively) mental health scores is shown in Figure 4. Poor mental health caused by the pandemic may offset the positive effect of distance learning on residents’ competencies. The highest level of competencies was found in individuals who reported less mental distress and adopted distance learning. Furthermore, the moderating effect of poor mental health on 4 subcompetencies (ie, MK-1, MK-2, SBP-2, and ICS) was similar to it on total competencies (*P*<.10; Multimedia Appendix 7).

**Table 3.** The moderating effects of mental health status on the association between competencies and distance learning.

Variables	Multiple linear regression models		
	β (SE)	90% CI	*P* value
Distance learning	0.63 (0.17)	0.34 to 0.91	<.001
Mental health status	–0.07 (0.06)	–0.17 to 0.04	.28
Interaction (distance learning*mental health status)	–0.15 (0.08)	–0.27 to –0.02	.06
Age (years) (reference ≤27)			
>27	0.12 (0.06)	0.01 to 0.22	.07
Sex (reference=male)			
Female	–0.27 (0.06)	–0.37 to –0.17	<.001
Education (reference=bachelor’s degree)			
Master’s or doctoral degree	0.19 (0.11)	0.01 to 0.38	.10
Training year (reference=second year)			
Third year	0.51 (0.06)	0.41 to 0.61	<.001
Working hours per week (reference ≤40 hours per week)			
40‐48	0.01 (0.07)	–0.12 to 0.13	.93
>48	0.01 (0.07)	–0.11 to 0.13	.86
Income	0.01 (0.01)	–0.01 to 0.03	.26
Type of residents (reference=nonprofessional master)			
Professional master	–0.03 (0.08)	–0.17 to 0.10	.70

## Discussion

### Principal Findings

Based on a national survey of radiology residents in China, our study found that radiology residents who received distance learning were more likely to report high proficiency in key competencies after 1 year. This was particularly true for learning knowledge and communication skills but was less evident in obtaining technical skills. In addition, we found a significant moderating effect of mental health status on the association between distance learning and competencies during COVID-19.

Web-based training programs are proposed to mitigate the loss of learning from clinical rotations during the pandemic [39,40]. Consistent with the results of previous findings that distance learning has the potential to improve learners’ academic performance, skill development, and engagement [39,41], our study observed a positive impact of distance learning on radiology residents’ self-assessed competencies. However, it should be noted that the impact of distance learning on professional competency varies among radiology residents. Our study enriches the understanding of distance learning by using milestones of 9 subcompetencies for 6 core competencies. We also assessed the long-term outcomes of distance learning among radiology residents in the initial stage of the COVID-19 pandemic (between January and May 2020). These findings can be used to help inform evidence-based policies to improve residency training in the future.

Knowledge gain is an important indicator of what trainees have learned during SRT training [42]. The radiology residents in our study reported a substantial gain of knowledge and communication skills during SRT, particularly in ICS competency and SBP competency that include clinical knowledge and medical humanities, with a regression coefficient >0.5. Whereas, the effect of distance learning on professional attitudes (PROF) and professional growth in clinical practice (PBLI) was moderate, with a regression coefficient between 0.3 and 0.4, which is in line with previous studies [43-45]. Distance learning was found to be supportive in the continuity of teaching and learning during COVID-19 [39], which might explain why residents who received distance learning reported a higher score in the competence of medical knowledge than nondistance learners. In addition, radiology residents are able to participate in web-based conferences and conduct digital medical consultations to enhance their competency of communication [43], though it could be hard to evaluate their body language in distance learning [46]. Our findings validated the positive role of distance learning in fulfilling the objectives of training by creating an enabling environment, especially in the domain of knowledge and communication. In other words, distance learning can help keep the consistency of residency programs during pandemics, facilitating the resilience and recovery of health systems.

Our findings showed that competence in procedures (PC-2) benefited the least from distance learning, followed by competencies in technical skills (including PC competencies that reflect radiology residents’ professionalism and MK competencies that reflect mastery of professional knowledge and imaging technology). The result is consistent with previous findings that most medical students feel unable to acquire practical clinical skills through web-based teaching during COVID-19 [47]. This could be explained by the reduction in daily cases due to COVID-19, which was one of the deficiencies in distance learning [20]. To address this challenge, new technologies such as touchscreen Anatomage Table and Touch Surgery application have been used to strengthen trainings of plastic surgery [48]. These new approaches can be integrated into web-based residency training to compensate for the shortage of training in technical skills with distance learning. Other training programs, such as competence in procedures, could also be carried out among radiology residents in the post–COVID-19 era.

Health professional education involves maintaining a sense of purpose and mental well-being (eg, balance work life, stress, depression, burnout, and more) among residents [49-51]. Our study extended the prior results by identifying that poor mental health moderates the relationship between distance learning and competency among radiology residents. During the COVID-19 pandemic, physicians experienced more mental health disorders [52] with negative impacts (eg, higher turnover intention and lower work performance) [53,54]. However, good mental health may help trainees to receive distance learning consistently [55]. When trainees are in a suboptimal mental status, they tend to have negative attitudes toward learning and are discouraged from receiving distance learning continuously [55]. Health workforce is the backbone of health systems in response of pandemics [56]. In addition to the structural changes required for the improvement of health systems’ resilience, it could also be necessary to provide long-term psychological support for the health workforce to help them overcome psychological distress [57]. In summary, several recommendations can be drawn from this study: (1) distance learning can be used for transferring knowledge to residents particularly when challenges exist in the traditional offline approaches; (2) clinical skills have a crucial role in offline training, which should be noted and well used by training institutions; (3) instructors could use a scientific and caring approach with a special focus on learners’ psychological well-being when preparing material and organizing courses; and (4) physicians who have engaged in distance learning could enhance their learning experience through an enhancement of their offline learning environment. This promotes a better psychological state for learning to become more effective.

Health system resilience is critical in training the required competencies of the health workforce [49]. In addition to competency-based education, the COVID-19 pandemic highlighted the application of digital interprofessional education [58]. In the future, distance learning may help physicians gain knowledge and skills in public health, interprofessional communication, and teamwork. In this regard, remote residency training should be developed as a holistic educational concept rather than a mere substitution for traditional in-person learning. Distance learning enables physicians to have a flexible schedule, a feasible access to classes, and an opportunity to keep a good balance between work and life [14]. What is more, the facilitation of distance learning contributes to the sharing of high-quality educational resources in the post–COVID-19 era. In particular, for health professionals in resource-constrained places (eg, rural areas in western China), the provision of high-quality distance learning could help them overcome geographical constraints and thus reduce inequalities in access to education [49,59]. Nevertheless, it is not possible to replace in-person teaching with distance learning completely. An integrated model of the 2 is encouraged to maximize the advantages of different teaching modalities.

### Limitations

This study has several limitations. First, we used SEM for the self-evaluation of radiology residents’ competencies; results may be influenced by the Dunning-Kruger effect, where participants with lower abilities tend to overestimate their competencies and the potential self-reporting bias [60]. Second, the lower response rate may be subject to selection bias, as our survey is voluntary in nature. However, based on sample size calculations [61], we obtained 1573 valid samples (precision=5%; baseline proportion=0.50), which accurately represent the characteristics of the residents. Importantly, this represents the largest nationally representative sample of radiology residents in China to date, which may help to minimize the potential bias. Third, although mental health status was asked by a single question based on the self-report psychological distress on a Likert scale, this variable is correlated with long-term mental health status (depression and burnout). Other potential moderating factors that may influence learning status, such as the design of the web-based course, courseware, and teaching styles, could be explored in future research. Fourth, the generalization of the results is another limitation of our study [49].

### Future Directions

As distance learning is anticipated to be applied across various fields, additional research is warranted to substantiate our findings. Longitudinal studies are recommended for future research to fully assess the long-term effects of distance learning on competence and mental health.

### Conclusions

Distance learning helps mitigate the negative impact of the COVID-19 lockdown on the education of health professionals. Meanwhile, attention should be paid to the disadvantages of distance learning and the mental health status of learners, as they may negatively influence the effectiveness and sustainability of distance learning. Our study provides insights into the role of distance learning in residency training during the pandemic.

## Supplementary material

10.2196/54228 Multimedia Appendix 1 The Spearman *r* between short- and long-term mental health status.

10.2196/54228 Multimedia Appendix 2 Self-evaluation milestone scores for radiology residents between distance learning and nondistance learning.

10.2196/54228 Multimedia Appendix 3 Distance learning efforts for radiology residents who had not participated in COVID-19–related activities.

10.2196/54228 Multimedia Appendix 4 Associations between distance learning and competencies among residents.

10.2196/54228 Multimedia Appendix 5 Association between distance learning and the overall average self-evaluation milestone score among residents.

10.2196/54228 Multimedia Appendix 6 The Spearman *r* between mental health status and competencies.

10.2196/54228 Multimedia Appendix 7 The moderating effects of mental health on the association between subcompetencies and distance learning.

## Abbreviation

ACGME: Accreditation Council of Graduate Medical Education
CAR: Chinese Association of Radiologists
ICS: interpersonal communication skill
MK: medical knowledge
MLR: multiple linear regression
PBLI: practice-based learning and improvement
PC: patient care
PROF: professionalism
SBP: system-based practice
SEM: self-evaluation milestone
SRT: standardized residency training

## References

[1] Zhang P, Gao J (2021). Evaluation of China’s public health system response to COVID-19. J Glob Health.

[2] Liu Q, Luo D, Haase JE (2020). The experiences of health-care providers during the COVID-19 crisis in China: a qualitative study. Lancet Glob Health.

[3] Xu TL, Ao MY, Zhou X (2020). China’s practice to prevent and control COVID-19 in the context of large population movement. Infect Dis Poverty.

[4] Li Z, Chen Q, Feng L (2020). Active case finding with case management: the key to tackling the COVID-19 pandemic. Lancet.

[5] Dong C, Tian Y, Xu W (2020). Introduction on collective quarantine of close contacts of patients with COVID-19 for medical observation in China: from the perspective of frontline staff. Biosci Trends.

[6] Bourgeault IL, Maier CB, Dieleman M (2020). The COVID-19 pandemic presents an opportunity to develop more sustainable health workforces. Hum Resour Health.

[7] Kuhlmann E, Dussault G, Wismar M (2020). Health labour markets and the “human face” of the health workforce: resilience beyond the COVID-19 pandemic. Eur J Public Health.

[8] Kruk ME, Myers M, Varpilah ST, Dahn BT (2015). What is a resilient health system? Lessons from Ebola. Lancet.

[9] Zhang J, Han X, Yang Z (2021). Radiology residency training in China: results from the first retrospective nationwide survey. Insights Imaging.

[10] National Health and Family Planning Commission, State Commission Office for Public Sector Reform (SCOPSR), National Development and Reform Commission, Ministry of Education, Ministry of Finance, Ministry of Human Resources and Social Security, State Administration of Traditional Chinese Medicine (2013). Guiding opinions on establishing the standardised residency training system. National Health Commission of the People’s Republic of China.

[11] (2019). Diagnostic radiology milestones. The Accreditation Council for Graduate Medical Education.

[12] Nasca TJ, Philibert I, Brigham T, Flynn TC (2012). The next GME accreditation system—rationale and benefits. N Engl J Med.

[13] Leddy R, Lewis M, Ackerman S (2017). Practical implications for an effective radiology residency quality improvement program for milestone assessment. Acad Radiol.

[14] Chen D, Ayoob A, Desser TS, Khurana A (2022). Review of learning tools for effective radiology education during the COVID-19 era. Acad Radiol.

[15] Dedeilia A, Sotiropoulos MG, Hanrahan JG, Janga D, Dedeilias P, Sideris M (2020). Medical and surgical education challenges and innovations in the COVID-19 era: a systematic review. In Vivo.

[16] Lo HY, Lin SC, Chaou CH, Chang YC, Ng CJ, Chen SY (2020). What is the impact of the COVID-19 pandemic on emergency medicine residency training: an observational study. BMC Med Educ.

[17] Brady A, Brink J, Slavotinek J (2020). Radiology and value-based health care. JAMA.

[18] Cavalieri S, Spinetta M, Zagaria D (2021). The impact of COVID-19 pandemic on radiology residents in Northern Italy. Eur Radiol.

[19] Alvin MD, George E, Deng F, Warhadpande S, Lee SI (2020). The impact of COVID-19 on radiology trainees. Radiology.

[20] Patil NS, Gunter D, Larocque N (2022). The impact of the COVID-19 pandemic on radiology resident education: where do we go from here?. Acad Radiol.

[21] Larocque N, Shenoy-Bhangle A, Brook A, Eisenberg R, Chang YM, Mehta P (2021). Resident experiences with virtual radiology learning during the COVID-19 pandemic. Acad Radiol.

[22] Lanier MH, Wheeler CA, Ballard DH (2021). A new normal in radiology resident education: lessons learned from the COVID-19 pandemic. Radiographics.

[23] Chénard-Roy J, Guitton MJ, Thuot F (2021). Online residency training during the COVID-19 pandemic: a national survey of otolaryngology head and neck surgery program directors. J Otolaryngol Head Neck Surg.

[24] Awan OA (2021). Virtual radiology readouts after the coronavirus disease (COVID-19) pandemic. AJR Am J Roentgenol.

[25] Chen Q, Liang M, Li Y (2020). Mental health care for medical staff in China during the COVID-19 outbreak. Lancet Psychiatry.

[26] Giusti L, Mammarella S, Salza A (2021). Predictors of academic performance during the covid-19 outbreak: impact of distance education on mental health, social cognition and memory abilities in an Italian university student sample. BMC Psychol.

[27] Zhao L, Hwang WY, Shih TK (2021). Investigation of the physical learning environment of distance learning under COVID-19 and its influence on students’ health and learning satisfaction. Int J Distance Educ Technol.

[28] Williamson DL, Carr J (2009). Health as a resource for everyday life: advancing the conceptualization. Crit Public Health.

[29] Airila A, Hakanen JJ, Schaufeli WB, Luukkonen R, Punakallio A, Lusa S (2014). Are job and personal resources associated with work ability 10 years later? The mediating role of work engagement. Work Stress.

[30] Raja U, Azeem MU, Haq IU, Naseer S (2020). Perceived threat of terrorism and employee outcomes: the moderating role of negative affectivity and psychological capital. J Bus Res.

[31] Stirpe L, Profili S, Sammarra A (2022). Satisfaction with HR practices and employee performance: a moderated mediation model of engagement and health. Eur Manag J.

[32] Di Malta G, Bond J, Conroy D, Smith K, Moller N (2022). Distance education students’ mental health, connectedness and academic performance during COVID-19: a mixed-methods study. Distance Educ.

[33] Lyle B, Borgert AJ, Kallies KJ, Jarman BT (2016). Do attending surgeons and residents see eye to eye? An evaluation of the Accreditation Council for Graduate Medical Education milestones in general surgery residency. J Surg Educ.

[34] Barbato KBG, de Carvalho LS, Barreira Marangoni V, de Souza F, de Vasconcelos Vaena MM (2023). Core competencies self-assessment and patient-practitioner orientation during the first year of a Brazilian orthopedic residency. Rev Bras Ortop (Sao Paulo).

[35] Watson RS, Borgert AJ, O׳Heron CT (2017). A multicenter prospective comparison of the Accreditation Council for Graduate Medical Education milestones: clinical competency committee vs. resident self-assessment. J Surg Educ.

[36] Kwasny L, Shebrain S, Munene G, Sawyer R (2021). Is there a gender bias in milestones evaluations in general surgery residency training?. Am J Surg.

[37] Li Z, Chen L, Li M, Cohen J (2018). Prenatal exposure to sand and dust storms and children’s cognitive function in China: a quasi-experimental study. Lancet Planet Health.

[38] Hodkinson A, Zhou A, Johnson J (2022). Associations of physician burnout with career engagement and quality of patient care: systematic review and meta-analysis. BMJ.

[39] Ahmady S, Kallestrup P, Sadoughi MM (2021). Distance learning strategies in medical education during COVID-19: a systematic review. J Educ Health Promot.

[40] Chick RC, Clifton GT, Peace KM (2020). Using technology to maintain the education of residents during the COVID-19 pandemic. J Surg Educ.

[41] Abdull Mutalib AA, Md Akim A, Jaafar MH (2022). A systematic review of health sciences students’ online learning during the COVID-19 pandemic. BMC Med Educ.

[42] Ritzmann S, Hagemann V, Kluge A (2014). The Training Evaluation Inventory (TEI)—evaluation of training design and measurement of training outcomes for predicting training success. Vocat Learn.

[43] Warnica W, Moody A, Probyn L, Bartlett E, Singh N, Pakkal M (2021). Lessons learned from the effects of COVID-19 on the training and education workflow of radiology residents—a time for reflection: perspectives of residency program directors and residents in Canada. Can Assoc Radiol J.

[44] Biswas SS, Biswas S, Awal SS, Goyal H (2022). Current status of radiology education online: a comprehensive update. SN Compr Clin Med.

[45] Liao F, Murphy D, Wu JC, Chen CY, Chang CC, Tsai PF (2022). How technology-enhanced experiential e-learning can facilitate the development of person-centred communication skills online for health-care students: a qualitative study. BMC Med Educ.

[46] Reinhart A, Malzkorn B, Döing C, Beyer I, Jünger J, Bosse HM (2021). Undergraduate medical education amid COVID-19: a qualitative analysis of enablers and barriers to acquiring competencies in distant learning using focus groups. Med Educ Online.

[47] Dost S, Hossain A, Shehab M, Abdelwahed A, Al-Nusair L (2020). Perceptions of medical students towards online teaching during the COVID-19 pandemic: a national cross-sectional survey of 2721 UK medical students. BMJ Open.

[48] Zingaretti N, Contessi Negrini F, Tel A, Tresoldi MM, Bresadola V, Parodi PC (2020). The impact of COVID-19 on plastic surgery residency training. Aesth Plast Surg.

[49] Frenk J, Chen LC, Chandran L (2022). Challenges and opportunities for educating health professionals after the COVID-19 pandemic. Lancet.

[50] Rotenstein LS, Berwick DM, Cassel CK (2022). Addressing well-being throughout the health care workforce: the next imperative. JAMA.

[51] Aiken LH, Simonetti M, Sloane DM (2021). Hospital nurse staffing and patient outcomes in Chile: a multilevel cross-sectional study. Lancet Glob Health.

[52] Li W, Frank E, Zhao Z (2020). Mental health of young physicians in China during the novel coronavirus disease 2019 outbreak. JAMA Netw Open.

[53] Al-Mansour K (2021). Stress and turnover intention among healthcare workers in Saudi Arabia during the time of COVID-19: can social support play a role?. PLoS One.

[54] Sadovyy M, Sánchez-Gómez M, Bresó E (2021). COVID-19: how the stress generated by the pandemic may affect work performance through the moderating role of emotional intelligence. Pers Individ Dif.

[55] Gu Z, Li P, Zhang A, Xu X, Gu F (2022). The role of mental health and sustainable learning behavior of students in education sector influences sustainable environment. Front Psychol.

[56] Witter S, Wurie H, Chandiwana P (2017). How do health workers experience and cope with shocks? Learning from four fragile and conflict-affected health systems in Uganda, Sierra Leone, Zimbabwe and Cambodia. Health Policy Plan.

[57] Dean L, Cooper J, Wurie H (2020). Psychological resilience, fragility and the health workforce: lessons on pandemic preparedness from Liberia and Sierra Leone. BMJ Glob Health.

[58] Samarasekera DD, Nyoni CN, Amaral E, Grant J (2022). Challenges and opportunities in interprofessional education and practice. Lancet.

[59] Huang Y (2020). Research on online education in the midst of the COVID-19 pandemic. JAER.

[60] Kruger J, Dunning D (1999). Unskilled and unaware of it: how difficulties in recognizing one’s own incompetence lead to inflated self-assessments. J Pers Soc Psychol.

[61] Hu LP, Liu HG (2005). Triple-type theory of statistics and its application in the scientific research of biomedicine. Zhonghua Yi Xue Za Zhi.

