# Supplementary material for: Distance Learning During the COVID-19 Lockdown and Self-Assessed Competency Development Among Radiology Residents in China: Cross-Sectional Survey
Source: JMIR Med Educ. 2025 May 8;11:e54228. doi: 10.2196/54228 (PMC12080970; doi:10.2196/54228)
Supplement: Multimedia Appendix 1 [file mededu-v11-e54228-s001.pdf]

	Total (n=2381)	Distance Learning (n=1699)	Non-distance Learning (n=682)
Depression	0.260***	0.265***	0.230***
Burnout	0.249***	0.263***	0.204***

Note: *p < 0.05; **p < 0.01; ***p<0.05. The Spearman's r was calculated to examine the association between short-term mental health status and long-term mental health status (depression and burnout).
