# Supplementary material for: Distance Learning During the COVID-19 Lockdown and Self-Assessed Competency Development Among Radiology Residents in China: Cross-Sectional Survey
Source: JMIR Med Educ. 2025 May 8;11:e54228. doi: 10.2196/54228 (PMC12080970; doi:10.2196/54228)
Supplement: Multimedia Appendix 2 [file mededu-v11-e54228-s002.pdf]

| **Diagnostic Radiology Subcompetencies** | **Total (N=2381)** | **Distance learning** | | ***P*-value** |
|---|---|---|---|---|
| | | **Yes (n=1699)** | **No (n=682)** | |
| **PC** | | | | |
| PC-1: Image Interpretation | 3.90±1.69 | 3.96±1.68 | 3.75±1.72 | .006 |
| PC-2: Competence in Procedures | 2.25±1.77 | 2.28±1.81 | 2.16±1.65 | .23 |
| **MK** | | | | |
| MK-1: Diagnostic Knowledge | 3.75±1.75 | 3.82±1.76 | 3.59±1.73 | .004 |
| MK-2: Imaging Technology and Image Acquisition | 3.53±1.90 | 3.62±1.92 | 3.29±1.81 | <.001 |
| **SBP** | | | | |
| SBP-1: System navigation for patient-centered care | 2.86±1.88 | 2.96±1.90 | 2.61±1.80 | <.001 |
| SBP-2: Contrast agent safety | 3.57±1.95 | 3.71±1.98 | 3.21±1.80 | <.001 |
| **PBLI** | | | | |
| PBLI: Evidence-Based and Informed Practice | 3.25±1.84 | 3.34±1.86 | 3.02±1.78 | <.001 |
| **PROF** | | | | |
| PROF: Self-Awareness and Help Seeking | 3.49±1.90 | 3.61±1.92 | 3.20±1.83 | <.001 |
| **ICS** | | | | |
| ICS: Patient- and Family-centered Communication | 3.72±2.10 | 3.87±2.11 | 3.33±2.03 | <.001 |
| **Average (all subcompetencies)** | 3.37±1.47 | 3.46±1.49 | 3.13±1.39 | <.001 |
