# Supplementary material for: Distance Learning During the COVID-19 Lockdown and Self-Assessed Competency Development Among Radiology Residents in China: Cross-Sectional Survey
Source: JMIR Med Educ. 2025 May 8;11:e54228. doi: 10.2196/54228 (PMC12080970; doi:10.2196/54228)
Supplement: Multimedia Appendix 3 [file mededu-v11-e54228-s003.pdf]

Diagnostic Radiology Subcompetencies	Residents have participated in COVID-19-related activities					
	Yes (N=853)			No (N=1528)		
	Distance Learning (n=610)	Non-distance Learning (n=243)	*P*-value	Distance Learning (n=1089)	Non-distance Learning (n=439)	*P*-value
**PC**						
PC-1: Image Interpretation	4.20±1.74	3.91±1.71	0.027	3.83±1.63	3.67±1.72	.10
PC-2: Competence in Procedures	2.55±2.01	2.44±1.82	0.453	2.14±1.68	2.00±1.52	.12
**MK**						
MK-1: Diagnostic Knowledge	4.00±1.81	3.90±1.85	0.490	3.72±1.72	3.42±1.64	.002
MK-2: Imaging Technology and Image Acquisition	3.98±1.97	3.70±1.94	0.058	3.42±1.87	3.07±1.69	<.001
**SBP**						
SBP-1: System navigation for patient-centered care	3.23±1.97	3.01±1.99	0.143	2.80±1.84	2.38±1.65	<.001
SBP-2: Contrast agent safety	4.00±2.01	3.64±1.92	0.015	3.55±1.95	2.97±1.69	<.001
**PBLI**						
PBLI: Evidence-Based and Informed Practice	3.60±1.85	3.49±1.90	0.461	3.20±1.84	2.76±1.66	<.001
**PROF**						
PROF: Self-Awareness and Help Seeking	3.83±1.92	3.50±1.83	0.023	3.49±1.90	3.04±1.81	<.001
**ICS**						
ICS: Patient- and Family-Centered Communication	4.20±2.09	3.69±2.12	0.001	3.69±2.09	3.13±1.96	<.001
**Average (all subcompetencies)**	3.73±1.52	3.47±1.47	0.025	3.31±1.45	2.94±1.32	<.001
