# Supplementary material for: Distance Learning During the COVID-19 Lockdown and Self-Assessed Competency Development Among Radiology Residents in China: Cross-Sectional Survey
Source: JMIR Med Educ. 2025 May 8;11:e54228. doi: 10.2196/54228 (PMC12080970; doi:10.2196/54228)
Supplement: Multimedia Appendix 4 [file mededu-v11-e54228-s004.pdf]

| Diagnostic Radiology Subcompetencies | Multiple Linear Regression Models | | |
|---|---|---|---|
| | *β (SE)* | *90% CI* | *P* value |
| **PC** | | | |
| PC-1: Image Interpretation | | | |
| Distance learning (ref.= No) | 0.23 (0.08) | (0.11, 0.35) | .002 |
| PC-2: Competence in Procedures | | | |
| Distance learning (ref.= No) | 0.14 (0.08) | (0.01, 0.26) | .09 |
| **MK** | | | |
| MK-1: Diagnostic Knowledge | | | |
| Distance learning (ref.= No) | 0.24 (0.08) | (0.11, 0.37) | .002 |
| MK-2: Imaging Technology and Image Acquisition | | | |
| Distance learning (ref.= No) | 0.34 (0.08) | (0.20, 0.48) | <.001 |
| **SBP** | | | |
| SBP-1: System navigation for patient-centered care | | | |
| Distance learning (ref.= No) | 0.36 (0.08) | (0.22, 0.50) | <.001 |
| SBP-2: Contrast agent safety | | | |
| Distance learning (ref.= No) | 0.52 (0.09) | (0.38, 0.67) | <.001 |
| **PBLI** | | | |
| PBLI: Evidence-Based and Informed Practice | | | |
| Distance learning (ref.= No) | 0.33 (0.08) | (0.19, 0.47) | <.001 |
| **PROF** | | | |
| PROF: Self-Awareness and Help Seeking | | | |
| Distance learning (ref.= No) | 0.41 (0.09) | (0.27, 0.55) | <.001 |
| **ICS** | | | |
| ICS: Patient- and Family-Centered Communication | | | |
| Distance learning (ref.= No) | 0.55 (0.09) | (0.39, 0.70) | <.001 |
| **Average (all subcompetencies)** | | | |
| Distance learning (ref.= No) | 0.35 (0.07) | (0.24, 0.45) | <.001 |

***Note:*** All models were controlled for participants' characteristics (age, gender, educational level, training years, working hours per week, annual after-tax income in 2020, and type of residents).
