# Supplementary material for: Distance Learning During the COVID-19 Lockdown and Self-Assessed Competency Development Among Radiology Residents in China: Cross-Sectional Survey
Source: JMIR Med Educ. 2025 May 8;11:e54228. doi: 10.2196/54228 (PMC12080970; doi:10.2196/54228)
Supplement: Multimedia Appendix 5 [file mededu-v11-e54228-s005.pdf]

Variables	Multiple Linear Regression Models		
	β (SE)	***90% CI***	***P*-value**
Distance learning	0.35 (0.07)	(0.24, 0.45)	<.001
Age (ref. ≤27)			
>27	0.12 (0.06)	(0.01, 0.22)	.07
Gender (ref. Male)			
Female	-0.28 (0.06)	(-0.37, -0.18)	<.001
Education (ref. Bachelor)			
Master or doctoral degree	0.18 (0.12)	(-0.01, 0.37)	.12
Training year (ref. 2nd year)			
3rd year	0.51 (0.06)	(0.41, 0.61)	<.001
Working hours per week (ref. ≤40hours/week)			
40-48	-0.002 (0.07)	(-0.12, 0.12)	.98
>48	-0.02 (0.07)	(-0.14, 0.10)	.83
Income	0.01 (0.01)	(-0.01, 0.03)	.25
Type of residents (ref. non-professional master)			
Professional master	-0.02 (0.08)	(-0.16, 0.11)	.77
