# Supplementary material for: Distance Learning During the COVID-19 Lockdown and Self-Assessed Competency Development Among Radiology Residents in China: Cross-Sectional Survey
Source: JMIR Med Educ. 2025 May 8;11:e54228. doi: 10.2196/54228 (PMC12080970; doi:10.2196/54228)
Supplement: Multimedia Appendix 6 [file mededu-v11-e54228-s006.pdf]

	Total (n=2381)	Distance Learning (n=1699)	Non-distance Learning (n=682)
**PC**			
PC-1: Image Interpretation	-0.058**	-0.070**	-0.023
PC-2: Competence in Procedures	-0.020	-0.002	-0.056
**MK**			
MK-1: Diagnostic Knowledge	-0.080**	-0.098**	-0.028
MK-2: Imaging Technology and Image Acquisition	-0.055**	-0.069**	-0.011
**SBP**			
SBP-1: System navigation for patient-centered care	-0.067**	-0.073**	-0.045
SBP-2: Contrast agent safety	-0.084**	-0.094**	-0.040
**PBLI**			
PBLI: Evidence-Based and Informed Practice	-0.068**	-0.081**	-0.024
**PROF**			
PROF: Self-Awareness and Help Seeking	-0.108**	-0.112**	-0.083*
**ICS**			
ICS: Patient- and Family-Centered Communication	-0.128**	-0.139**	-0.085*
**Average (all subcompetencies)**	-0.098**	-0.107**	-0.059

Note: *P < .05; **P < .01. The Spearman's r was calculated to examine the association between mental health status and competencies.
