# Supplementary material for: Distance Learning During the COVID-19 Lockdown and Self-Assessed Competency Development Among Radiology Residents in China: Cross-Sectional Survey
Source: JMIR Med Educ. 2025 May 8;11:e54228. doi: 10.2196/54228 (PMC12080970; doi:10.2196/54228)
Supplement: Multimedia Appendix 7 [file mededu-v11-e54228-s007.pdf]

Variables	Multiple Linear Regression Models		
	β (SE)	*90% CI*	*P* value
**PC-1: Image Interpretation**			
Distance learning	0.47 (0.20)	(0.15, 0.80)	.02
Mental health status	-0.02 (0.07)	(-0.14, 0.10)	.77
Interaction (Distance learning*Mental health status)	-0.13 (0.09)	(-0.27, 0.02)	.17
**PC-2: Competence in Procedures**			
Distance learning	0.10 (0.21)	(-0.24,0.44)	.62
Mental health status	-0.06 (0.08)	(-0.18, 0.07)	.45
Interaction (Distance Learning*Mental health status)	0.01 (0.09)	(-0.14, 0.17)	.89
**MK-1: Diagnostic Knowledge**			
Distance learning	0.67 (0.21)	(0.33, 1.01)	.001
Mental health status	-0.01 (0.08)	(-0.14 ,0.11)	.85
Interaction (Distance Learning*Mental health status)	-0.22 (0.09)	(-0.38, -0.07)	.02
**MK-2: Imaging Technology and Image Acquisition**			
Distance learning	0.67 (0.22)	(0.31, 1.04)	.003
Mental health status	-0.03 (0.08)	(-0.16, 0.11)	.74
Interaction (Distance Learning*Mental health status)	-0.17 (0.10)	(-0.34, -0.01)	.09
**SBP-1: System navigation for patient-centered care**			
Distance learning	0.66 (0.22)	(0.30, 1.02)	.003
Mental health status	-0.06 (0.08)	(-0.19, 0.07)	.47
Interaction (Distance Learning*Mental health status)	-0.16 (0.10)	(-0.32, 0.01)	.12
**SBP-2: Contrast agent safety**			
Distance learning	0.88 (0.23)	(0.51, 1.25)	<.001
Mental health status	-0.05 (0.08)	(-0.19, 0.09)	.54
Interaction (Distance Learning*Mental health status)	-0.19 (0.10)	(-0.36, -0.02)	.07
**PBLI: Evidence-Based and Informed Practice**			
Distance learning	0.62 (0.22)	(0.26, 0.97)	.004
Mental health status	-0.03 (0.08)	(-0.16, 0.10)	.67
Interaction (Distance Learning*Mental health status)	-0.15 (0.10)	(-0.31, 0.01)	.13
**PROF: Self-Awareness and Help Seeking**			
Distance learning	0.59 (0.22)	(0.23, 0.96)	.008
Mental health status	-0.17 (0.08)	(-0.31, -0.04)	.03
Interaction (Distance Learning*Mental health status)	-0.10 (0.10)	(-0.27, 0.06)	.31
**ICS: Patient- and Family-Centered Communication**			
Distance learning	0.96 (0.24)	(0.56, 1.36)	<.001
Mental health status	-0.17 (0.09)	(-0.32, -0.02)	.06
Interaction (Distance Learning*Mental health status)	-0.22 (0.11)	(-0.40, -0.04)	.05

Abbreviations: All models were controlled for participants' characteristics (age, gender, educational level, training years, working hours per week, annual after-tax income in 2020, and types of residents).
